# Autophagy Modulation in Cancer Immunotherapy, Emerging Molecular Targets and Drug Selection Strategies

**DOI:** 10.3390/ijms27052183

**Published:** 2026-02-26

**Authors:** Maroua Jalouli, Abdel Halim Harrath, Mohammed Al-Zharani, Md Ataur Rahman

**Affiliations:** 1 Department of Biology, College of Science, Imam Mohammad Ibn Saud Islamic University (IMSIU), Riyadh 11623, Saudi Arabia; mejalouli@imamu.edu.sa (M.J.); mmylzahrani@imamu.edu.sa (M.A.-Z.); 2 Department of Zoology, College of Science, King Saud University, Riyadh 11451, Saudi Arabia; hharrath@ksu.edu.sa; 3 Department of Oncology, Karmanos Cancer Institute, Wayne State University, Detroit, MI 48201, USA

**Keywords:** autophagy modulation, cancer immunotherapy, molecular targets, tumor microenvironment, drug selection strategies, combination therapy

## Abstract

Cancer immunotherapy has revolutionized the treatment of cancer by harnessing the immune system to recognize and destroy malignant cells. However, a substantial proportion of patients exhibit primary or acquired resistance to these therapies, underscoring the urgent need to identify novel molecular targets to enhance therapeutic efficacy. Autophagy, an evolutionarily conserved cellular process of degradation and recycling, has emerged as a critical modulator of tumor immunity and the function of immune cells. In cancer cells, autophagy modulates antigen presentation, immunogenic cell death, metabolic reprogramming, and resistance to immune-mediated cell death. Concurrently, autophagy rigorously governs the viability, differentiation, and functional capacity of immune cells, including T cells, dendritic cells, macrophages, and natural killer (NK) cells. Dysfunctional autophagic flux in the tumor microenvironment can enhance immune evasion and limit the efficacy of immune checkpoint inhibitors, adoptive cell therapies, and cancer vaccines. In this review, we provide an in-depth analysis of emerging molecular targets involved in the regulation of autophagy relevant to cancer immunotherapy. This includes key signaling pathways such as PI3K/AKT/mTOR, AMPK, Beclin-1 complexes, ULK1, and lysosomal regulators. Additionally, we explore the rational integration of the pharmacological modulation of autophagy, including small molecules, natural compounds, and nanoparticle-based drug delivery systems, with immunotherapeutic approaches. We highlight the importance of rational drug selection and combination therapies to overcome resistance to immunotherapy and minimize toxicity. Understanding the context-dependent role of autophagy will be essential for the development of next-generation, precision-targeted cancer immunotherapies. Therefore, a comprehensive understanding of the context-specific functions of autophagy in tumor and immune cells is crucial for devising precision-targeted combination methods that overcome immunotherapy resistance and produce more sustainable cancer treatment outcomes.

## 1. Introduction

Immunotherapy represents a milestone in current cancer treatment, conferring durable and even curative responses across a broad spectrum of malignancies [1]. Immune checkpoint blockade, adoptive cell therapy, and cancer vaccines have provided a significant therapeutic benefit by reinvigorating anti-tumor immune surveillance [2]. However, despite these promising advances, a large proportion of patients remain unresponsive or eventually develop resistance. Tumor-intrinsic evolution, immune cell dysfunction, and immunosuppressive tumor microenvironment (TME) collectively contribute to the limited effectiveness of immunotherapy, highlighting the urgent need for new molecular targets that can restore immune sensitivity and improve therapeutic outcomes.

Autophagy is a highly conserved catabolic process that degrades and recycles damaged organelles, misfolded proteins, and metabolic substrates to maintain cellular homeostasis [3,4]. In the context of cancer, autophagy plays a dual role that is highly dependent on the tumor stage and microenvironment [5]. While basal autophagy may act as a tumor suppressor by mitigating genomic instability and oxidative stress, established tumors often exploit elevated autophagic flux to survive under conditions of nutrient deprivation, hypoxia, and therapeutic stress [6]. Notably, emerging evidence indicates a profound connection between autophagy and cancer immunity. Autophagic flux in tumor cells can influence antigen processing and presentation, immunogenic cell death (ICD), cytokine secretion, and metabolic reprogramming, all of which directly impact immune recognition and effector function [7].

In addition to tumor cells, autophagy plays a critical role in the biology of immune cells. T cells require tightly regulated autophagy for their survival, differentiation, and memory formation [8]. Dendritic cells require functional autophagic machinery for efficient antigen presentation and cross-priming. In macrophages and natural killer (NK) cells, autophagy mediates inflammatory signaling, cytotoxicity, and polarization [9]. Autophagy dysregulation in immune populations can also compromise anti-tumor immunity and contribute to immune exhaustion, leading to resistance to immunotherapy [10]. The complex TME further complicates this relationship by subjecting tumor and immune cells to metabolic and oxidative stress, which in turn alters autophagic signaling [11]. Hypoxia, nutrient deprivation, and chronic inflammation can drive maladaptive autophagy, contributing to immune evasion and therapy failure. Therefore, autophagy has emerged as an attractive yet complex therapeutic target for improving cancer immunotherapy.

In this review, we would like to discuss the emerging molecular targets involved in the regulation of autophagy for cancer immunotherapy. The objective of this review is to propose a framework for leveraging autophagy modulation to overcome immunotherapy resistance and improve precision cancer therapy by integrating molecular understanding with therapeutic strategies, such as rational drug selection and combination strategies.

## 2. Molecular Mechanisms of Autophagy in Tumor Cells Versus Immune Cells

Autophagy is a highly regulated lysosomal degradation process that executes selective, context-dependent functions in both tumor and immune cells. Despite the conservation of the core autophagic machinery across different cell types, including the ULK1 initiation complex, Beclin-1-VPS34 nucleation complex, ATG conjugation systems, and lysosomal fusion machinery, the upstream signaling pathways and downstream consequences of autophagy activation are distinct between the malignant and immune compartments [12,13].

**Figure 1:**
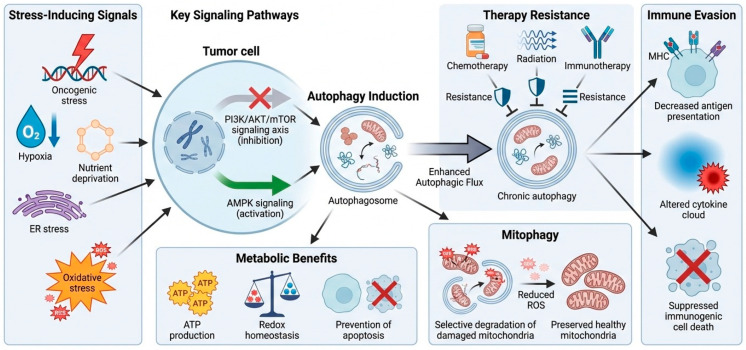
**Molecular mechanisms of autophagy in tumor cells.** Stress-induced autophagy in tumor cells and its implications for treatment resistance and immune evasion. Oncogenic stress, hypoxia, nutrient deprivation, endoplasmic reticulum stress, and oxidative stress inhibit PI3K/AKT/mTOR signaling and activate AMPK, leading to the induction of autophagosome formation and increased autophagic flux. Chronic autophagy enables metabolic adaptation, redox homeostasis, mitophagy-mediated mitochondrial quality control, and evasion of apoptosis, ultimately contributing to the development of resistance to chemotherapy, radiotherapy, and immunotherapy, as well as decreased antigen presentation, immunogenic cell death, and antitumor immune recognition.

In tumor cells, autophagy frequently serves as a cytoprotective process that promotes metabolic flexibility and therapy resistance. Oncogenic stress, hypoxia, nutrient deprivation, endoplasmic reticulum stress, and oxidative stress robustly induce autophagy through inhibiting the PI3K/AKT/mTOR signaling axis and activating AMPK signaling [14]. Enhanced autophagic flux allows tumor cells to recycle intracellular components to fuel ATP production, maintain redox homeostasis, and prevent apoptosis in hostile environments [11]. Autophagy supports the selective degradation of damaged mitochondria through mitophagy, limiting the excessive accumulation of reactive oxygen species (ROS) and maintaining mitochondrial integrity [15]. In advanced tumors, chronic autophagy activation can contribute to immune evasion by reducing surface antigen presentation, modulating cytokine secretion, and limiting immunogenic cell death [16]. As a result, tumor cell-intrinsic autophagy is often associated with resistance to chemotherapy, radiation, and immunotherapy (Figure 1).

**Figure 2:**
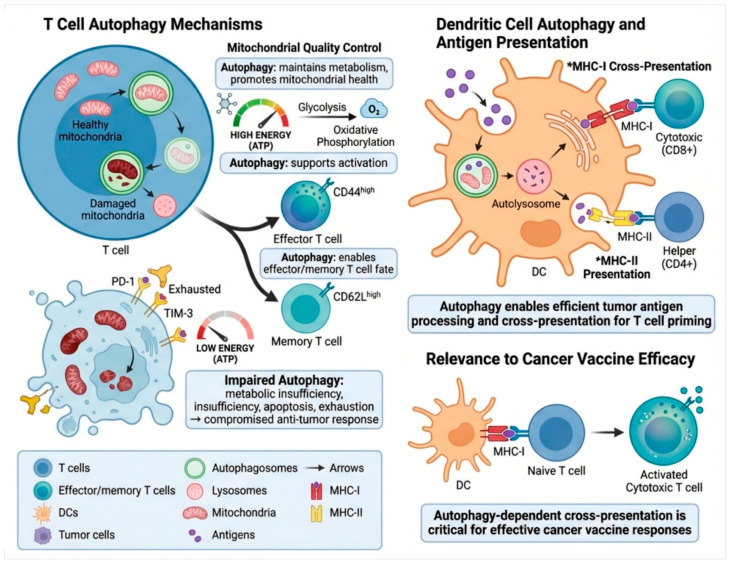
**Molecular mechanisms of autophagy in immune cells.** The key functions of autophagy for immune cell homeostasis, activation, and antitumor immunity are described. In T cells, autophagy sustains mitochondrial quality and metabolic fitness during effector and memory differentiation while preventing exhaustion. In dendritic cells, autophagy allows efficient tumor antigen processing and MHC-I and MHC-II presentation, thereby supporting CD8^+^ and CD4^+^ T-cell priming. In addition, autophagy-dependent cross-presentation is required for potent immune activation and cancer vaccine efficacy. * MHC-II signifies a crucial, highly regulated component of the immune system.

Immune cell autophagy is an evolutionarily conserved homeostatic and regulatory process that plays a key role in promoting immune activation, metabolic fitness, and antitumor immunity, as depicted in Figure 2. Autophagy maintains basal intracellular quality control mechanisms in T cells by selectively degrading damaged mitochondria through mitophagy, which restricts the excessive production of reactive oxygen species and preserves mitochondrial function [17]. During T-cell activation, autophagy facilitates metabolic reprogramming to support T-cell proliferation and effector functions by mediating the metabolic switch between glycolysis and oxidative phosphorylation to fuel high bioenergetic demands [18]. In this context, autophagy plays a crucial role in the differentiation of naïve T cells into effector cells and memory T-cell subsets with antitumor functions, which is important for the maintenance of antitumor immunity and long-term responses to immunotherapy [19]. Impaired autophagic flux in T cells leads to the accumulation of dysfunctional mitochondria, resulting in ATP depletion, oxidative stress, and increased susceptibility to apoptosis [20]. These metabolic defects contribute to T-cell exhaustion, which is characterized by the upregulation of inhibitory receptors, such as PD-1 and TIM-3, and impaired cytokine production. Therefore, cytotoxic activity against tumor cells is reduced, resulting in impaired immune-mediated tumor clearance.

In dendritic cells, autophagy plays a central role in antigen processing and presentation [9]. Fusion of autophagosomes with lysosomes results in the efficient degradation of tumor-derived antigens and their loading onto both MHC class I and class II molecules, supporting cross-presentation to CD8^+^ cytotoxic T lymphocytes and classical presentation to CD4^+^ helper T cells to orchestrate adaptive immune responses [21]. Autophagy-dependent cross-presentation is also a key determinant of effective T-cell priming and is required for the efficacy of cancer vaccines [22]. Taken together, Figure 2 shows that intact autophagy in immune cells underlies immune homeostasis, optimizes antigen presentation, and promotes antitumor immunity, and underscores the importance of maintaining immune cell autophagy when considering autophagy-modulating cancer therapies.

Macrophages and natural killer (NK) cells exhibit autophagy-dependent modulation of inflammatory signaling, polarization, and cytotoxic functions. In macrophages, autophagy influences the balance between pro-inflammatory M1 and immunosuppressive M2 phenotypes, thereby shaping immune responses in the TME [23]. In NK cells, autophagy regulates the homeostasis of granzyme and perforin, thereby maintaining the ability to kill tumor cells [24]. As a result, autophagy elicits opposing biological consequences in tumor cells versus immune cells, promoting survival and immune evasion in cancer cells while supporting immune cell function and anti-tumor effector capacity [16]. Understanding these distinct molecular processes is essential for developing therapeutic strategies that selectively inhibit autophagy in tumor cells while preserving or enhancing autophagy in immune cells to improve cancer immunotherapy outcomes.

## 3. Autophagy in the Tumor Microenvironment and Immune Evasion

The tumor microenvironment (TME) is a complex milieu composed of malignant cells, immune infiltrates, stromal cells, extracellular matrix, and soluble factors [25,26]. In tumor cells, microenvironmental cues that trigger autophagy support cellular adaptation and survival during metabolic stress, while also dampening antitumor immune responses [27]. Enhanced autophagic flux leads to the degradation of immunogenic cellular components, reduced surface expression of tumor antigens, and impaired release of damage-associated molecular patterns (DAMPs) required for immunogenic cell death [28]. Autophagy in non-malignant components of the TME also contributes to immune suppression. Tumor-associated macrophages (TAMs) with elevated autophagic activity often acquire an immunosuppressive M2-like phenotype, characterized by the secretion of IL-10 and TGF-β, which restrict cytotoxic T-cell functions [29]. In cancer-associated fibroblasts (CAFs), autophagy enables metabolic coupling with tumor cells by providing recycled nutrients, indirectly promoting tumor growth and contributing to immunological dysfunction.

**Figure 3:**
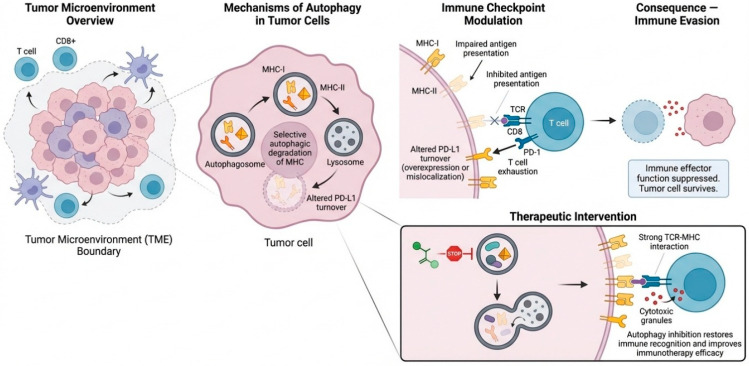
**Autophagy in the tumor microenvironment and immune evasion.** The role of tumor cell–intrinsic autophagy in sculpting the immune-privileged microenvironment that tumors require to grow. Autophagy driven by metabolic stress conditions leads to selective degradation of MHC class I and II as well as altered turnover of PD-L1. The combination of these leads to defective antigen presentation and increased PD-L1 signaling to dampen the T-cell response. This impairs TCR-MHC binding, leads to T-cell exhaustion, and inhibits cytotoxic effector responses, leading to tumor survival. Autophagy inhibition reverses these effects to restore antigen presentation, normalize checkpoint signaling, and reactivate CD8^+^ T-cell–mediated killing and sensitizes tumors to cancer immunotherapy.

In the tumor microenvironment, autophagy directly regulates immune checkpoint expression, antigen presentation, and T cell function (Figure 3). Autophagy can specifically target and degrade major histocompatibility complex (MHC) class I and class II molecules, reducing the presentation of tumor antigens on the cell surface [21]. This downregulation of antigen presentation impairs T-cell receptor (TCR) recognition and leads to diminished cytotoxic T-cell activation [30]. Additionally, autophagy modulates immune checkpoint interactions, such as those involving PD-L1. Dysregulated autophagy can alter PD-L1 turnover, either stabilizing it at the tumor cell surface or causing its mislocalization, which enhances the inhibitory PD-1 signaling in T cells [31]. This results in decreased cytokine production, reduced cytotoxic granule release, and T-cell exhaustion.

In the tumor microenvironment, these autophagy-dependent mechanisms work in concert to dampen immune effector functions, allowing tumor cells to evade immune detection while maintaining resilience to therapeutic-induced cellular stress [32]. The dual role of autophagy in integrating metabolic adaptation with immune evasion mechanisms, thus becoming a major driver of resistance to immune checkpoint inhibitors [33]. Notably, inhibiting autophagy in tumor cells can restore antigen presentation, normalize immune checkpoint expression, and enhance TCR-MHC binding [34]. This reactivation of immune surveillance mechanisms can potentiate cytotoxic T-cell-mediated killing and improve the overall efficacy of immunotherapeutic strategies. Targeting evasion and in a tumor-selective manner within the tumor microenvironment is therefore an attractive approach to reversing immune suppression, limiting immune evasion, and sensitizing resistant tumors to cancer immunotherapy.

## 4. Drug Selection and Therapeutic Modulation of Autophagy to Enhance Immunotherapy in Cancer

Drug selection must align with the immunotherapy environment, the tumor’s reliance on autophagy, and the vitality of immune cells. In “autophagy addicted” cancers, inhibiting late-stage autophagy can reinstate antigenicity and enhance sensitivity to PD-1 and PD-L1 blocking. In alternative contexts, the induction of autophagy can diminish PD-L1 levels and enhance immunological priming [35].

### 4.1. Small-Molecule Drugs

The use of small-molecule autophagy modulators for combination with immunotherapy is very attractive since these agents can be readily scaled, often have oral bioavailability, and are amenable to combinations with checkpoint blockade or innate immune agonists [36] (Table 1). The challenge is knowing when to inhibit and when to induce autophagy. Late-stage lysosomal inhibitors (chloroquine, hydroxychloroquine) prevent autophagosome-lysosome fusion and may exacerbate tumor immunogenic stress; however, these agents can also disrupt T-cell functions under certain scenarios, and should be considered judiciously in terms of patient, schedule, and dosage [37]. Targeting upstream pathways of autophagy modulation can affect both tumor and immune compartments. mTOR inhibitors (rapalogs, such as temsirolimus, everolimus, rapamycin, sirolimus) can impact metabolism, antigen presentation, and PD-L1 biology through the mTOR-autophagy axis, and some studies have shown immune-related effects, such as modulation of extracellular vesicle PD-L1 [38]. Metabolic modulators such as metformin, which activate AMPK, induce autophagy, and reprogram tumor, stromal, and immune metabolism, have been viewed as an intuitive match for immunotherapy, particularly where metabolic dysfunction causes T-cell dysfunction [39]. In the end, autophagy inhibition can be achieved in a pathway-specific manner (e.g., Vps34) and can convert “cold” tumors to inflamed phenotypes and improve responses to PD-1 or PD-L1 blockade in preclinical settings, thus supporting a precision medicine approach: inhibit autophagy when it supports immune exclusion, and induce autophagy when it supports antigen presentation and immune function [40].

### 4.2. Bioactive Natural Products

Bioactive natural compounds can modulate autophagy through redox, metabolic, and stress-adaptation pathways that interface with immunological checkpoints, antigen presentation, and inflammatory signaling [49]. Many drugs are pleiotropic, which can support multi-node reprogramming of the tumor microenvironment; however, this comes at the cost of the need for patient stratification and mechanism anchoring [50]. A particularly relevant mechanism in the context of immunotherapy is the autophagy-mediated regulation of PD-L1 expression and trafficking [51]. Many drugs have been shown to promote selective autophagic degradation or downregulation of PD-L1, thereby boosting T-cell function and potentially potentiating checkpoint blockade response (Table 2). Curcumin is one of the most notable examples, with the literature describing its immunomodulatory effects and context-dependent actions in the tumor microenvironment, as well as changes in autophagy-related signaling [52]. Many polyphenols and alkaloids are often studied as regulators of AMPK, mTOR, NF-κB, and oxidative stress pathways that modulate autophagy flux and inflammatory cytokine profiles, which can impact dendritic cell priming, macrophage polarization, and T-cell exhaustion programs that are central barriers to immunotherapy efficacy [53,54]. From a pharmacological perspective, natural products are best deployed as adjuvants when they have a specified autophagy directionality that is relevant to the tumor type in question, a mechanistic link to immune evasion phenotypes such as PD-L1, antigen presentation, or immunosuppressive myeloid cells, and a viable delivery strategy, often enabled by nanoformulations to overcome poor bioavailability.

### 4.3. Synthetic Chemical Drugs

Synthetic drugs can target autophagy with high precision at a certain time point, such as ULK1 initiation, Vps34 nucleation, Beclin complex stability, or lysosomal degradative function [4]. Many of these agents have advantages over repurposed drugs in terms of potency and selectivity, which is particularly important in the setting of immunotherapy, where systemic inhibition of autophagy could harm immune cell function (Table 3). Examples of ULK1 inhibitors include SBI-0206965 and ULK-101. The inhibition of ULK1 prevents early autophagy initiation, and these inhibitors have been used extensively to validate ULK1 dependence for tumor cell survival [62]. Vps34 inhibitors, including SAR405, VPS34-IN1, and PIK-III, block PI3P production and autophagosome formation [63]. Targeting Vps34 is also linked to immune remodeling, such as the conversion of cold tumors into inflamed tumors, and priming PD-1 and PD-L1 blockade in preclinical models [64]. Additional lysosomal inhibitors beyond chloroquine include Lys05, ROC-325, and DQ661, which were designed to improve efficacy and overcome pharmacological limitations [65]. These drugs may cause tumor stress and, depending on the context, can also drive immune activation by increasing antigenicity or reducing immunosuppressive effects, but careful combination design is required to avoid interference with T-cell programs. Spautin-1 has a unique mechanism of disrupting Vps34 complexes via targeting deubiquitinase and the stability of Beclin1 complex and could potentially prime tumors to be more sensitive to additional treatment.

### 4.4. Nanoparticle-Based Drug Delivery

The administration of nanoparticles (NPs) can support the region- and time-dependent modulation of autophagy to strengthen immunotherapy and prevent systemic exposure that could threaten autophagy in immune cells [75]. Prominent approaches include: tumor-targeted delivery of autophagy inhibitors to increase immunogenic stress and/or enhance checkpoint blockade, immune-cell targeted delivery to reshape myeloid inhibition or improve antigen presentation, and the co-delivery of immuno-oncology drugs with agents that tune autophagy (Table 4). There is a strong rationale to use nanocarriers to improve the delivery of lysosomotropic autophagy inhibitors (e.g., chloroquine or hydroxychloroquine) to increase tumor accumulation and limit off-target side effects. Reviews note that targeting autophagy can increase the retention and efficacy of nanoparticles and that autophagy-modifying NPs can be rationally designed based on mTOR signaling, ROS, mitophagy, or fusion blocking [76]. Different systems offer an early-stage inhibitor such as 3-MA, or co-delivery of an autophagy modulator with chemotherapy, radiation sensitizers, or immunostimulants to trigger synchronized tumor stress and immune activation [77]. Recent reviews stress that nanomedicine can regulate autophagy in both tumor-associated and immune cells, which can be key to reprogramming suppressive tumor microenvironments (TMEs) to inflammatory and therapy-responsive states [78]. From a pharmacological standpoint, the most effective NP approach depends on whether autophagy must be blocked in tumor cells and/or preserved in T cells, and often favors tumor-targeted carriers, stimuli-responsive release (pH, ROS, enzyme), and combination treatments with PD-1/PD-L1 blockade, with timing tailored to avoid immune suppression [78].

Complete and total selectivity is not expected because immune cells and tumor cells use the same fundamental autophagy machinery, such as ULK1, Beclin-1–VPS34 complexes, ATG proteins, and lysosomal regulators. Nevertheless, functional selectivity can be attained by leveraging variations in cellular environment, activation state, and metabolic programming. Autophagy is closely linked to activation-induced metabolic reprogramming, mitochondrial quality control, and antigen presentation in immune cells, especially T cells and dendritic cells [87]. Autophagy in these cells is temporary, constantly changing, and mostly happens when the immune system is activated, not when the body is under long-term stress. Tumor cells, on the other hand, often depend on autophagy that is always high because of low oxygen levels, lack of nutrients, cancer-causing signals, and damage caused by treatment [88]. Therefore, inducing autophagy via immune-activating stimuli, such antigen exposure, cytokine signaling, or metabolic support, can selectively augment autophagy in immune cells while exerting minimal effects on tumor cells that are already functioning at near-maximal autophagic capacity [89]. Additionally, immune-directed delivery methods, such as manipulating immune cells outside the body, using vaccines to activate immune cells, or using metabolic modulators that work better on certain types of cells, make autophagy induction even more likely to happen in immune cells [90]. Consequently, although molecular pathways intersect, immune cell–specific autophagy stimulation can be attained via context- and activation-dependent processes.

## 5. Drug Selection and Rational Combination Strategies

Rational drug choices for targeting autophagy in the context of cancer immunotherapy should start with an accurate assessment of the dominant resistance mechanism at play, be it tumor cell-intrinsic immune resistance, myeloid suppressive programming, T-cell exhaustion, or defective antigen priming [91]. Autophagy underlies tumor cell immune resistance through stress buffering, attenuated signals of immunogenic cell death, and metabolic plasticity; at the same time, autophagy is crucial for immune cell function and antigen presentation [89]. Therefore, the overarching approach is one of selectivity, with the aim to dampen tumor-autophagy, while preserving or even boosting autophagy in effector cells. Patient and tumor stratification are critical for success. As such, a rational approach should assess baseline levels of autophagy activity (readouts like LC3-II, p62, and lysosomal genes); tumor immune contexture (degree of CD8 infiltration, myeloid skewing); metabolic and hypoxia signatures; and checkpoint biology (PD-L1 expression, interferon response signatures) [92]. In these cases, late inhibitors (lysosomal blockades) may increase tumor stress and immunogenicity, thereby sensitizing them to PD-1 or PD-L1 blockade [93]. ULK1 or Vps34 axis inhibitors may be preferred where lysosomal blockade is poorly tolerated, or a more targeted mechanism of action is needed.

The dominant approach to date has been that of priming then locking initially hit the brakes on the immune response with checkpoint inhibition or antigen-directed therapy, then bring in tumor-selective autophagy inhibition to prevent adaptive escape. Another approach is to stress-then-release with chemotherapy, radiation, or targeted agents that stress the tumor, then use autophagy inhibition to prevent repair and enable immune-mediated clearance [94]. The dose and schedule should avoid immune suppression, for instance via intermittent autophagy inhibition, tumor-selective dosing, and limiting peak inhibition at key phases of T-cell expansion [32]. Combination design must also consider other aspects of safety and tolerability such as overlapping myelosuppression, liver toxicity, and retinopathy risks from lysosomotropic drugs, and potential pharmacokinetic interactions. Finally, the monitoring of response should consider both immunological readouts such as T-cell reinvigoration and cytokine secretion profiles, and autophagy flux analyses to quickly adapt drug choice, timing, or dose intensification.

The selective inhibition of autophagy in tumor cells is more achievable than uniform systemic inhibition; nonetheless, it necessitates precision-oriented techniques instead of a comprehensive blockade. Tumor cells in the tumor microenvironment are more dependent on autophagy to stay alive when they are under constant metabolic and therapeutic stress [95]. This establishes a therapeutic window wherein partial or intermittent autophagy inhibition selectively undermines tumor survival while preserving the functional resilience of immune cells. Nanoparticles, antibody-drug conjugates, and pH- and enzyme-responsive formulations are examples of tumor-targeted delivery technologies that focus autophagy inhibitors in tumor tissue while limiting exposure to immune cells that are circulating [96]. Temporal separation techniques improve selectivity even more by stopping autophagy from happening during important times of immune cell growth and effector differentiation [97]. Importantly, immune cells can typically make up for short-term autophagy inhibition by changing their metabolism, but tumor cells cannot do this when they are under long-term stress.

## 6. Clinical Trial and Preclinical Modulation of Autophagy to Enhance Immunotherapy in Cancer

Both preclinical and early clinical studies substantiate the hypothesis that autophagy modification can affect the efficacy of cancer immunotherapy, however the depth and maturity of data vary significantly between these areas. Preclinical models offer robust mechanistic justification, illustrating that tumor cell–intrinsic autophagy enhances resistance to immune-mediated cytotoxicity by restricting immunogenic cell death, hindering antigen presentation, and stabilizing immune checkpoint signaling. In mice and in vitro models, pharmacological inhibition or genetic suppression of autophagy has demonstrated the capacity to augment T-cell infiltration, boost responses to PD-1 and PD-L1 blockage and sensitize tumors to combination immunotherapy regimens [98]. These studies have also shown that the state of autophagy in the tumor microenvironment affects how macrophages and dendritic cells work together and how tumor and immune cells compete for resources.

In contrast, clinical evidence remains limited and largely indirect. Early-phase clinical trials have mostly tested lysosomotropic autophagy inhibitors like chloroquine or hydroxychloroquine together with chemotherapy, radiation, or targeted treatments [99]. Immunotherapy objectives are frequently exploration rather than primary. Although many trials indicate acceptable safety profiles and moderate indications of increased tumor control, conclusive evidence confirming enhanced immunotherapy outcomes via autophagy modulation remains absent. It is significant that most trials have not categorized patients according to autophagy dependency or immunological contexture, complicating the identification of responding subgroups.

Furthermore, clinical translation is limited by difficulties in evaluating autophagy flux in patient samples. Dynamic assessment of autophagy in cancers and immune cells presents technical challenges in clinical settings, in contrast to controlled preclinical systems, hence restricting biomarker-guided treatment selection. Consequently, contemporary clinical trials frequently depend on empirical combination tactics instead than mechanism-driven approaches. In general, preclinical studies clearly support autophagy as a regulator of interactions between tumors and the immune system and as a possible target for immunotherapy [19]. Nonetheless, clinical validation is still in its infancy. Future trials that combine autophagy biomarkers, immune profiling, and smart combination techniques will be necessary to connect strong preclinical findings with immunotherapy improvements that can be used in the clinic.

## 7. Challenges and Limitations

A major challenge in directing autophagy to enhance cancer immunotherapy is the context-dependent and cell type-specific characteristics of autophagy [100]. The tumor heterogeneity itself poses yet another challenge. The tumor microenvironment also significantly contributes to this complexity through hypoxia, acidosis, nutrient deprivation, and myeloid-mediated suppression, all of which can affect autophagic flux and immune signaling [101]. Finally, the existing pharmacological tools in clinical use are still imperfect. Many of the widely used lysosomotropic inhibitors can have variable intratumoral exposures and off-target effects on lysosomes, and the newer more selective inhibitors are sometimes lacking in their safety data. There is also a lack of reliable tools to assess autophagy in patients, as static readouts such as LC3 or p62 are not always reflective of flux.

The preclinical and clinical data supporting the modulation of autophagy for the enhancement of immunotherapy is fraught with limitations. Many of the mechanistic understandings are based on models that may not fully capture the intricacies of the human immune landscape, including differences in myeloid composition, microbiome influences, and prior treatment exposures [102]. Additionally, the inconsistent use of autophagy flux assays remains a limitation. Static measurements of LC3-II accumulation or p62 levels can be misleading if not validated by dynamic flux measurements, especially in vivo [103]. Translating these findings into the clinic is challenging due to variable drug penetration into tumors, dose-limiting toxicities, and a lack of robust biomarkers for patient selection.

## 8. Future Perspectives

Future directions will include more strategic approaches to inform the when, where, and which cell types in which autophagy should be manipulated. A critical first step will be the development of robust biomarkers to assess autophagy flux in tumors and immune subsets from patients. Ideally, these would include multi-omics signatures, lysosome-targeted imaging probes, and serum markers of stress signaling. Nanoparticle- and antibody–drug conjugate approaches may also increase tumor penetration and allow for subcompartmental targeting, such as anti-tumor macrophages or the stromal microenvironment. Combination approaches will need to move beyond checkpoint inhibitors to include STING agonists, oncolytic viruses, vaccines, and adoptive cell therapy, in which autophagy manipulation may improve antigen presentation and immune priming. By linking autophagy biology to immune phenotypes and drug exposure, we can move towards precision therapeutics that can convert immune-deficient tumors into long-term responders.

## 9. Conclusions

Targeting autophagy represents an attractive strategy to overcome resistance to immune-based therapies by enhancing tumor antigenicity, modulating immune checkpoint balance, and reprogramming the immunosuppressive tumor microenvironment. Advances in small molecule inhibitors, bioactive natural products, and synthetic autophagy modulators have expanded the pharmacological toolkit. Furthermore, nanoparticle-mediated delivery systems show promise for tumor-selective modulation while minimizing systemic immune suppression. The careful selection of medications, dose optimization, and sequencing immunotherapies will be required to preserve immune function. Future therapeutic success will depend on the integration of reliable biomarkers of autophagy flux with immune profiling to select the patients most likely to respond to autophagy-targeted combinations.

## Figures and Tables

**Table 1:** Small-molecule modulators to modulate autophagy to enhance cancer immunotherapy.

Drug	Autophagy Action, Primary Node	Immunotherapy Relevance, Typical Rationale	References
Hydroxychloroquine (HCQ)	Late-stage inhibition, lysosomal deacidification	Sensitize tumors, combination trials with checkpoint blockade, chemo	[41]
Chloroquine (CQ)	Late-stage inhibition, autophagosome-lysosome fusion block	Tumor sensitization also impacts immune clearance of nanodrugs	[42]
Temsirolimus	mTOR inhibition, autophagy induction context-dependent	Can enhance anti-cancer immunity via autophagy-linked pathways	[38]
Everolimus	mTOR inhibition	Reported combinations with PD-1 blockades in preclinical settings	[43]
Rapamycin	mTOR inhibition	Immune modulation, T-cell metabolism, autophagy coupling	[44]
Sirolimus	mTOR inhibition	Trialed in combinations with immunomodulators	[45]
Metformin	AMPK Activation, autophagy induction	Metabolic reprogramming to support immunity, combination rationale	[39]
3-Methyladenine (3-MA)	Early-stage inhibition, PI3K/Vps34 related	Tool and preclinical sensitizer, often used in combo strategies	[46]
Spermidine	Autophagy induction	Immune-supportive autophagy induction discussed in immuno-oncology	[47]
Trehalose	Autophagy induction	Immune-supportive autophagy induction discussed in immuno-oncology	[48]

**Table 2:** Bioactive natural compounds to regulate autophagy for the enhancement of cancer immunotherapy.

Natural Product	Autophagy Linked to Mechanism, Immune Angle	Cancer Immunotherapy Rationale	References
Curcumin	Modulates autophagy signaling, immune microenvironment	Adjuvant to immunotherapy, immune reprogramming	[55]
Resveratrol	AMPK, mTOR, autophagy-linked stress control	Potentially improves immune fitness, reduces suppressive signaling	[56]
Quercetin	Redox and autophagy pathway modulation	May alter TME inflammation and antigenicity	[57]
Berberine	Metabolic stress pathways, autophagy modulation	Potential to reduce immune evasion programs	[58]
EGCG	Stress signaling, autophagy modulation	Immune supportive, anti-inflammatory balance	[59]
Andrographolide	Reported PD-L1 regulation via autophagy-related routes	Enhances anti-tumor immunity, PD-L1 axis	[35]
Sulforaphane	Autophagy and redox remodeling	Potentially improves immunogenicity and TME tone	[60]
Spermidine	Autophagy induction, immune function support	Improves immune competence, discussed as immuno-oncology support	[47]
Trehalose	Autophagy induction	Immune supportive autophagy induction	[48]
Artesunate	Stress responses, autophagy modulation	Combination rationale with immunotherapy via stress immunogenicity	[61]

**Table 3:** Synthetic autophagy-targeting agents to target cancer immunotherapy.

Drug	Target, Autophagy Node	Typical Use Case Relevant to Immunotherapy	References
SBI-0206965	ULK1 inhibitor, initiation block	Early autophagy inhibition, sensitization strategies	[66]
ULK-101	ULK1 inhibitor, initiation block	Potent ULK1 inhibition, preclinical validation	[67]
SAR405	Vps34 inhibitor, nucleation block	Autophagy inhibition, synergy with mTOR inhibitors	[68]
VPS34-IN1	Vps34 inhibitor	Selective Vps34 blockade, pathway dissection	[69]
PIK-III	Vps34 inhibitor	Acute autophagy inhibition tool, substrate discovery	[70]
Spautin-1	USP10/USP13, Beclin complex destabilization	Autophagy inhibition, chemosensitization frameworks	[71]
Lys05	Lysosomal autophagy inhibitor	More potent CQ derivative, tumor stress induction	[72]
ROC-325	Lysosomal autophagy inhibitor	Orally bioavailable, stronger than HCQ in models	[73]
DQ661	PPT1 inhibitor, lysosome targeting	Unified lysosomal targeting, blocks autophagy, mTOR	[74]
3-MA	PI3K related, early inhibition tool	Preclinical sensitizer, widely used experimental inhibitor	[46]

**Table 4:** Nanoparticle Approaches for Modulating Autophagy in Cancer Immunotherapy.

Nano-Strategy	Cargo, Target, Autophagy Intent	Immunotherapy Relevance	References
CQ-loaded nanoparticles	CQ, late-stage inhibition, tumor targeting	Enhance delivery and efficacy, reduce clearance	[79]
HCQ nanoformulation	HCQ, late-stage inhibition	Improve tumor accumulation, combination potential	[80]
3-MA polymeric nanoparticles	3-MA plus chemo, early-stage inhibition	Intensify tumor stress with controlled delivery	[81]
Autophagy-targeting nano-drug delivery systems	Multiple cargos, fusion blockades and lysosome targeting	Broad platform overview relevant to immunotherapy	[82]
Nanotherapeutics modulating mTOR autophagy axis	Rapalogs or pathway modulators	Combine autophagy modulation with tumor targeting	[83]
ROS-responsive autophagy-modulating nanoparticles	ROS-triggered release	Stress immunogenicity plus checkpoint synergy concept	[83]
Mitophagy-targeting nanomedicine	Mitochondria-directed cargos	Rewire tumor metabolism and immune visibility	[84]
Nanomedicine for autophagy modulation	Platform strategies across tumors and immune cells	Combination strategies, targeting immune suppression	[85]
Autophagy-targeting nanomedicine, PPT1 axis	DQ661-like lysosome targeting concept	Tumor stress, possible immune sensitization	[81]
Nanomaterial-based autophagy modulation	Engineered nanomaterials tuning autophagy	Design principles for tumor and immune cell targeting	[86]

## Data Availability

No new data were created or analyzed in this study. Data sharing is not applicable to this article.
